# Transcriptional patterns of *Coffea arabica* L. nitrate reductase, glutamine and asparagine synthetase genes are modulated under nitrogen suppression and coffee leaf rust

**DOI:** 10.7717/peerj.8320

**Published:** 2020-01-03

**Authors:** Viviane Yumi Baba, Masako Toma Braghini, Tiago Benedito dos Santos, Kenia de Carvalho, João Danillo Moura Soares, Suzana Tiemi Ivamoto-Suzuki, Mirian P. Maluf, Lilian Padilha, Luzia D. Paccola-Meirelles, Luiz Filipe Pereira, Douglas S. Domingues

**Affiliations:** 1 Department of Agronomy, Universidade Estadual de Londrina, Londrina, Paraná, Brazil; 2 Plant Biotechnology Laboratory, Instituto Agronômico do Paraná, Londrina, Paraná, Brazil; 3 Centro de Análise e Pesquisa Tecnológica do Agronegócio do Café “Alcides Carvalho,” Instituto Agronômico de Campinas, Campinas, São Paulo, Brazil; 4 Programa de Pós-Graduação em Agronomia, Universidade do Oeste Paulista, Presidente Prudente, São Paulo, Brazil; 5 Plant Biotechnology Laboratory, Embrapa Soja, Londrina, Paraná, Brazil; 6 Department of Botany, Instituto de Biociências, São Paulo State University, UNESP, Rio Claro, São Paulo, Brazil; 7 Plant Breeding, Embrapa Café, Brasília-DF, Brazil; 8 Department of Agronomy, Universidade Paranaense, Umuarama, Paraná, Brazil

**Keywords:** N assimilation, Gene expression, *Hemileia vastatrix*, Mineral nutrition, Genotypic response, RT-qPCR

## Abstract

This study evaluated the transcriptional profile of genes related to nitrogen (N) assimilation in coffee plants susceptible and resistant to rust fungi under N sufficiency and N suppression. For this purpose, we inoculated young coffee leaves with *Hemileia vastatrix* uredospores and collected them at 0, 12, 24 and 48 hours post-inoculation (HPI) to evaluate the relative expressions of genes encoding cytosolic *glutamine synthetase* (*CaGS_1_*), plastid *glutamine synthetase* (*CaGS_2_*), *nitrate reductase* (*CaNR*), and *asparagine synthetase* (*CaAS*). The genes exhibited distinct patterns of transcriptional modulation for the different genotypes and N nutritional regimes. The resistant genotype (I59) presented high levels of transcription in response to pathogen inoculation for *CaNR* and *CaGS_1_* genes, evaluated under N sufficiency in the initial moments of infection (12 HPI). The gene *CaGS_1_* also showed a peak at 48 HPI. The susceptible genotype (CV99) showed increased transcript rates of *CaNR* at 12 and 24 HPI in response to rust inoculation. The transcriptional patterns observed for CV99, under N suppression, were high levels for *CaAS* and *CaGS_2_* at all post-inoculation times in response to coffee leaf rust disease. In addition, *CaGS_1_* was up-regulated at 48 HPI for CV99. Cultivar I59 showed high transcript levels at 12 HPI for *CaAS* and peaks at 24 and 48 HPI for *CaGS_2_* in inoculated samples. Consequently, total chlorophyl concentration was influenced by N suppression and by rust infection. Regarding enzyme activities in vitro for glutamine synthetase and *CaNR*, there was an increase in infected coffee leaves (I59) and under N sufficiency. Moreover, CV99 was modulated in both N nutritional regimes for GS activity in response to rust. Our results indicate that N transport genes trigger a differential modulation between genotypes through the action of rust disease.

## Introduction

Coffee is one of the most important tropical commodities in the world, and Brazil is the largest producer and exporter of *Coffea arabica* L. (International Coffee Organization (ICO), 2018). Coffee farmers face serious problems due to crop susceptibility to diseases, which leads to a reduction in yield and to increased production costs. *C. arabica* plantations around the world are susceptible to numerous fungal diseases, of which coffee leaf rust (CLR), caused by the basidiomycete *Hemileia vastatrix* Berk & Br, is one of the most aggressive ones. This fungus mainly attacks coffee tree leaves, causing losses in the coffee production of around 35–50% (Zambolim et al., 2005). Forty-five physiological races of *H. vastatrix* are currently known (Várzea & Marques, 2005), and 15 of these have already been reported in Brazil (Zambolim et al., 2005; Cabral et al., 2009; Capucho et al., 2012).

Nitrogen (N) is an essential macronutrient for plants, playing an important role in defense mechanisms against diseases (Krapp, 2015; Larbat et al., 2016; Bogaert et al., 2017; Roca et al., 2018). Generally, higher amounts of N fertilizer increase plant susceptibility to biotrophic pathogens, while the opposite can occur in necrotrophic pathogens, indicating that there is a complex relationship between N uptake and metabolism as well as disease infection (Fagard et al., 2014). Nitrogen availability and N sources (NO_3_^−^ or NH_4_^+^) can also affect the plant responses to diseases, depending on the species and the type of pathogen (Pageau et al., 2006; Gupta et al., 2013).

Nitrogen use includes uptake, assimilation, translocation, and remobilization processes (Masclaux-Daubresse et al., 2010). Glutamine, nitrate, and asparagine are the main amino acids involved in N translocation of many plant species (Tegeder & Masclaux-Daubresse, 2018; Moison et al., 2018).

In some pathosystems, it has been demonstrated that genes related to N metabolism have their transcriptional activity modulated, as in the case of anthracnose and common bean interaction (Tavernier et al., 2007), corn smut and maize (Horst et al., 2010), and bacterial spot and pepper (Hwang, An & Hwang, 2011). In tobacco plants under pathogen infection, the N nutrition status can affect the disease severity and the transcriptional patterns of genes involved in N metabolism (Pageau et al., 2006; Gupta et al., 2013). However, few studies have reported the transcriptional modulation of genes related to mineral nutrient assimilation and protection against pathogens, especially in tropical and perennial crops, such as coffee trees.

Molecular responses in the rust fungi and coffee interaction are usually studied using large-scale analyses (Fernandez et al., 2004, 2012; Florez et al., 2017) or identifying candidate genes related to plant–pathogen resistance (Ganesh et al., 2006; Ramiro et al., 2009; Diola et al., 2013). One of the most up-regulated genes modulated by rust infection in coffee plants is *asparagine synthetase* (*AS*) (Fernandez et al., 2012), supporting the hypothesis that N metabolism can influence plant resistance to this disease. *AS* has been reported during plant–pathogen interactions involved in the N remobilization in tomato, pepper, and *Arabidopsis* (Olea et al., 2004; Ward et al., 2010; Hwang, An & Hwang, 2011; Seifi et al., 2014). It has been suggested that during the pathogen attack, N remobilization occurs to redirect plant nutrients to the pathogen, supporting its growth, or to initiate plant defense responses by exporting its nutrients to uninfected organs (Pageau et al., 2006; Horst et al., 2010; Mur et al., 2017).

Within this context, the aim of this study was to evaluate the transcriptional activity of genes related to N assimilation during N-sufficiency and -suppression treatments in leaves infected with *H. vastatrix*, using susceptible and resistant coffee cultivars. Genes encoding glutamine synthetase (*CaGS*_*1*_ and *CaGS*_*2*_), nitrate reductase (*CaNR*), and *CaAS* had their transcriptional profile evaluated by real-time quantitative PCR (RT-qPCR) analysis. In addition, we quantified glutamine synthetase and *CaNR* in vitro activities and chlorophyl concentration in infected coffee leaves. This is the first study to provide valuable information on molecular responses involved in the N assimilation of coffee plants in response to CLR, the main disease that affects coffee production.

## Materials and Methods

### Plant material

Two coffee genotypes were used in this study: *C. arabica* L. cv. IAPAR59 (I59) and Catuaí Vermelho IAC 99 (CV99), considered resistant and susceptible to rust fungi, respectively (Sera et al., 2010; Del Grossi et al., 2013). Approximately 6-month-old plants were obtained from the coffee nursery of the “Instituto Agronômico do Paraná” (IAPAR–latitude 23°18′ S, longitude 51°09′ O, average elevation 585 m; Londrina, Paraná, Brazil). Forty-two plants of each cultivar, with uniform size and development, were selected and transferred to the greenhouse with a constant temperature of 25 °C. Plants were grown in 4.5 L-pots containing sterilized sand and maintained in an aerated solution modified from Clark (1975) at pH 5.5–6.0, containing 5,333 µm NH_4_NO_3_, 266 µm KH_2_PO_4_, 332.5 µm MgSO_4_, 1,068 µm K_2_SO_4_, 665 µm CaCl_2_, 200 µm Na-Fe-EDTA, 7 µm H_3_BO_3_, 3 µm MnSO_4_, 2.5 µm ZnSO_4_, 0.1 µm CuSO_4_, and 0.7 µm Na_2_MoO_4_.

### Induction of differential nutritional patterns

We applied different nutritional regimens for a period of 6 weeks. Plants under N sufficiency conditions were weekly irrigated with nutrient solution as above mentioned. Plants under N suppression were treated with nutrient solutions without N sources. After this treatment, total N was quantified in leaves at the same developmental status, as described by Tang et al. (2012) and Dos Santos et al. (2019).

### CLR inoculation

The inoculum was composed of uredospores of *H. vastatrix* race II. We inoculated the CLR following the procedures described previously (Barsalobres-Cavallari et al., 2009). Experiments were conducted at the Coffee Center “Alcides Carvalho”, (Instituto Agronômico de Campinas–IAC, Campinas, Brazil). A uredospore suspension with a final concentration of one mg/mL was inoculated with an air compressor in young, fully expanded leaves. Inoculation was performed in four pairs of leaves per plant (first and second pair), in a total of 28 plants. As experimental control, plant leaves were mock-inoculated (water). To induce germination of urediospores in *H. vastatrix*, the coffee plants were kept in the greenhouse; subsequently, we transferred these plants to a humid chamber, covered them with black plastic, and maintained them in the darkness for 24 h at 22 °C and high relative humidity. Inoculated plants of the resistant and susceptible genotypes were maintained until the occurrence of symptoms to confirm fungus infection.

For biochemical and molecular experiments, plant leaves at the same developmental stage were collected after 0, 12, 24 and 48 hours post-inoculation (HPI). Leaves were collected, immediately frozen by immersion in liquid nitrogen, and stored at −80 °C for further analysis. The experimental design was completely randomized, with biological triplicates represented by pools of leaves from seven plants.

### Identification of genes related to N metabolism

Expressed sequence tags (ESTs) coding for cytosolic glutamine synthetase (*CaGS*_*1*_), plastid glutamine synthetase (*CaGS*_*2*_), *CaNR* and *CaAS* (Fernandez et al., 2012) were selected and downloaded from the database of the Brazilian Coffee Genome Project (Mondego et al., 2011). Additionally, homologous sequences of *Arabidopsis thaliana* were used as queries to identify genes, whose accesses are detailed and listed in Table 1. *Arabidopsis* protein sequences were submitted to the Blast program (tBlastN), using ESTs of *Coffea* spp. dbEST (NCBI) and CafEST (Mondego et al., 2011) as databases. We also included homologs from the *C. canephora* genome (Denoeud et al., 2014).

**Table 1:** Genes and primers used for RT-qPCR analysis.

Gene	Abbreviation	*C. arabica* contig	NCBI accession	*C. canephora* locus	*Arabidopsis* ortholog	Amplicon (pb)	Primer forward and reverse	Efficiency (%)
Glyceraldehyde-3-phosphate dehydrogenase	*CaGAPDH*	*	GW488886.1	Cc05_g16480	AT1G13440	100	F-AGGCTGTTGGGAAAGTTCTTC R-ACTGTTGGAACTCGGAATGC	94
Cytosolic glutamine synthetase	*CaGS*_*1*_	9226	GW485208.1	Cc07_g13290	AT1G66200.3	147	F-GAGGATCACTGAGATTGCTGGG R-TCAATTGCCTTCTTGATGACTCC	96
Plastid glutamine synthetase	*CaGS*_*2*_	9035	GR998899.1	Cc07_g13290	AT5G35630.3	143	F-ACTTCAAAGATGTGGAGCTCCC R-GTTACGTCCAGGTTTAGCAGCTGT	93
Nitrate reductase	*CaNR*	7146	GT687366.1	Cc00_g12040	AT1G77760.1	153	F-TGTGCTACGGTTGATGAAAAGCTAT R-ATCCAAGTATTGTGACATAACCCCTC	93
Asparagine synthetase	*CaAS*	6506	GW450683.1	Cc01_g14420	AT5G10240.2	101	F-GATTGATGACTGATGTGCCTTTTG R-GGCCTTTGTACCAGCCAAGTAA	93

**Note:**

* Based in Cruz et al. (2009).

### RNA isolation and cDNA synthesis

Total RNA was isolated as described by Korimbocus et al. (2002), using CTAB precipitation buffer associated with lithium chloride. Total RNA suspended in nuclease-free water was stored at −80 °C. All samples were treated with DNase I (RNase-free, Invitrogen^®^, Carlsbad, CA, USA) and the absence of genomic DNA contamination was verified by PCR using *GAPDH* primers (Table 1). The RNA integrity of all samples was assessed by analysis in 1% agarose gel, and A260/280 nm ratios were determined using a NanoDrop™ 1000 spectrophotometer (Thermo Fisher Scientific, Waltham, MA, USA). All samples had a A260/A280 nm ratio of 1.8–2.0, suitable for analysis. Complementary DNA (cDNA) was synthesized using SuperScript III Reverse Transcriptase (Invitrogen^®^, Carlsbad, CA, USA) following the manufacturer’s instructions in a final volume of 13 µL, using 2.5 µg of total RNA.

### Primer design and RT-qPCR analysis

Specific primers were designed using the Primer Express software v. 3.0 (Applied Biosystem, Foster City, CA, USA), aimed to obtain amplicons of 100–150 bp with an annealing temperature of 60 ± 2 °C (Table 1). To confirm the specificity of each primer pair, amplification products were verified in 2% agarose gel, stained with ethidium bromide, and photographed to ensure their size in accordance with the expectation.

The transcriptional abundance of N metabolism genes was analyzed by RT-qPCR, performed in a 7,500 Fast Real-Time PCR System (Applied Biosystems, Foster City, CA, USA) using the kit 2x SYBR Green/ROX qPCR Master Mix (Thermo Scientific, Waltham, MA, USA) according to the manufacturer’s instructions. Each reaction was performed in a volume of 25 μL, with 12.5 μL SYBR Green/ROX qPCR Master Mix, 0.5 μL primer (10 μm), 10.5 μL nuclease-free water, and one μL cDNA (five ng/μL). The amplification conditions were 10 min for 95 °C, followed by 40 cycles of amplification 95 °C for 30 s and 60 °C for 60 s. The amplification specificity of each reaction was verified and determined by dissociation curve analysis.

Relative expression levels of the genes were analyzed using the (1 + efficiency)^−ΔΔCt^ method (Livak & Schmittgen, 2001), where ΔCt_target_ = Ct_target_ _gene_ − Ct*_CaGAPDH_* and ΔΔCt = ΔCt_target gene_ − ΔCt_reference sample_. Coffee leaves for mock inoculation at 0 HPI were used as calibrator sample. Transcriptional levels were normalized using the *CaGAPDH* gene as reference gene, recommended by Barsalobres-Cavallari et al. (2009) and Cruz et al. (2009). We analyzed only the data obtaining at least 90% of amplification efficiency, estimated by the LinReg tool (Ramakers et al., 2003). All reactions were performed with three biological and technical replicates, following the MIQE guidelines for RT-qPCR experiments (Bustin et al., 2013).

### Biochemical analyses

#### Glutamine synthetase in vitro activity

The GS activity was determined according to the hydroximate biosynthetic methodology described by Cruz et al. (2004), also performed by Pompelli et al. (2010) in coffee leaves. The GS activity was measured using a UV–VIS spectrophotometer UV-1650PC (Shimadzu, Kyoto, Japan), quantifying the production of y-glutamyl-hydroxamate (540 nm).

#### Nitrate reductase in vitro activity

The NR activity was measured and adapted according to several protocols (Radin, 1973; Chow, De Oliveira & Pedersén, 2004; Reis et al., 2009). Plant tissue was ground in seven mL extraction buffer composed of HEPES–NaOH (pH 7.5) 100 mmol·L^−1^, MgCl_2_ 20 mmol·L^−1^, dithiothreitol 5 mmol·L^−1^, ascorbic acid 10 mmol·L^−1^, EDTA 5 mmol·L^−1^, 2% polyvinylpolypyrrolidone, FAD 10 µm, and leupeptine 10 µm. The reaction was incubated for 5 min in ice, and subsequently, the samples were centrifuged for 15 min at 4 °C. The supernatant was kept on ice and in the dark. After this, 400 µL extract were added to two mL of reaction buffer solution (HEPES–NaOH 50 mM·L^−1^ pH 7.5, MgCl_2_ 20 mM·L^−1^, DTT one mM·L^−1^, FAD 10 µm, KNO_3_ 10 mM·L^−1^, and NADH 0.2 mM·L^−1^). Samples without the addition of NADH were used as reaction control. The samples were kept in a water bath at 30 °C for 30 min in the dark, and the reaction was stopped with the addition of 150 µL zinc sulfate 0.4 M. Subsequently, we added 750 µL of 1% sulfanilamide solution in 3 M HCl and 750 µL of 0.02% of N-(1-naphthyl) ethylenediamine dihydrochloride (NED; m:v). After 2 min, the samples were centrifuged at 3,000*g* for 2 min, and the absorbance was evaluated in a spectrophotometer at 540 nm. Absorbance readings were performed in triplicate using one mL of reaction solution, applying a standard curve of NaNO_2_. The enzymatic activity was calculated in µM NO_2_/g based linear regression, using NaNO_2_ concentrations between 0 and 10 µmol·L^−1^, and presented a correlation coefficient (*r*^2^) over 0.99.

### Chlorophyll concentration

Chlorophyll concentration, chlorophyl *a*, and chlorophyl *b* were determined in leaf extracts following the protocol described by Arnon (1949). We used 100 mg of macerated coffee leaves in a 100% acetone extract with three biological replicates. All reactions were performed in the dark for 30 min and then centrifuged at 7,500 rpm for 10 min. A three mL aliquot was measured using a UV–VIS spectrophotometer UV-1650PC (Shimadzu, Kyoto, Japan) at 645, 663, and 652 nm.

### Statistical analysis

Data from RT-qPCR and biochemical analysis were submitted to analysis of variance (ANOVA) to determine differences between treatments at *p* < 0.05. Means were separated according to Tukey’s test (*p* < 0.05). All statistical analyses were performed using the ExpDes package (Ferreira, Cavalcanti & Nogueira, 2018) in the R software (R Core Team, 2018).

## Results

### Induction of the different nutritional patterns and CLR inoculation

The Fig. S1 shows the total N in leaves under N sufficiency, with values of 33.43 and 31.13 g·kg^−1^ for I59 and CV99, respectively. Plants under N suppression had their N content reduced and presented 24.95 g·kg^−1^ (I59) and 26.74 g·kg^−1^ (CV99). These results confirm the different plants nutritional status.

Figure S2 shows the pictures of susceptible plants (CV99) inoculated with *H. vastatrix*, used as control to confirm rust infection, with symptoms after 45 days post-inoculation. The coffee plant I59, considered a resistant cultivar, showed typical responses of an incompatible reaction. The pictures in the Fig. S3 show chlorotic spots with no fungus sporulation a typical hypersensitive response of resistant coffee leaves.

### Transcriptional analysis of genes involved in N assimilation process

**Figure 1:**
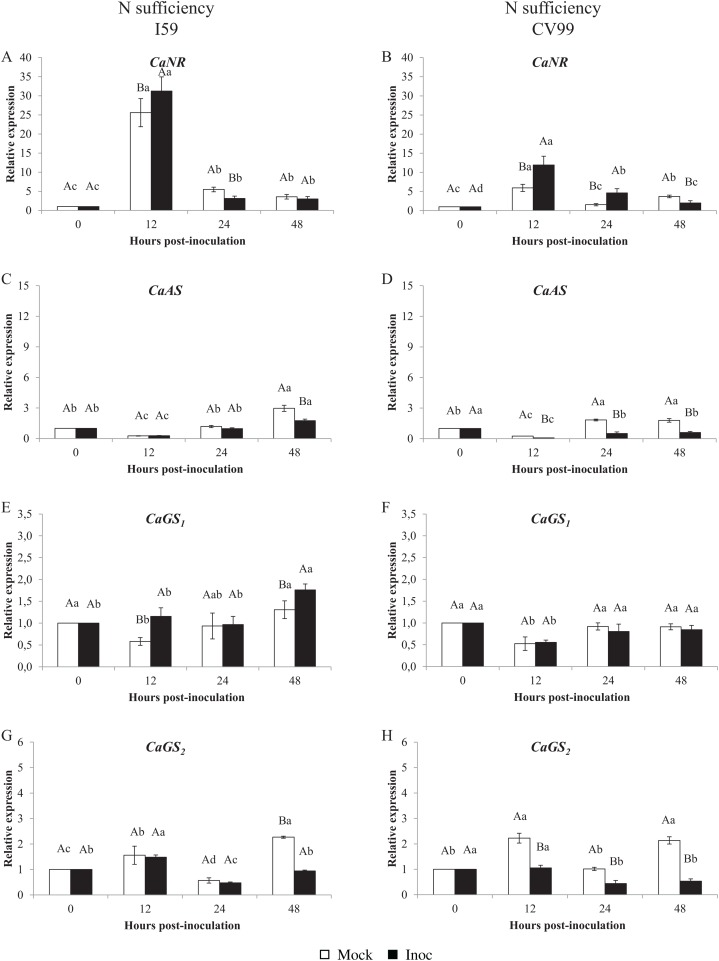
N metabolism gene expression patterns using RT-qPCR analysis for resistant (I59) (A, C, E and G) and susceptible (CV99) (B, D, F and H) genotypes under N sufficiency at 0, 12, 24 and 48 h post-inoculation with mock and rust inoculated coffee leaves. The vertical bars indicate the values presented by the mean ± standard error (*n* = 9) of N metabolism gene for each time post-inoculation and genotype. Averages followed by the same capital letter, compare differences between mock and rust inoculated coffee leaves at the same HPI, did not differ significantly by the Tukey test (*p* < 0.05). Averages followed by the same lowercase letter, compare differences between the treatments (mock and inoc) at different HPI, did not differ significantly by the Tukey test (*p* < 0.05). *CaGAPDH* was used as reference gene and 0 HPI mock inoculation as calibrator.

**Figure 2:**
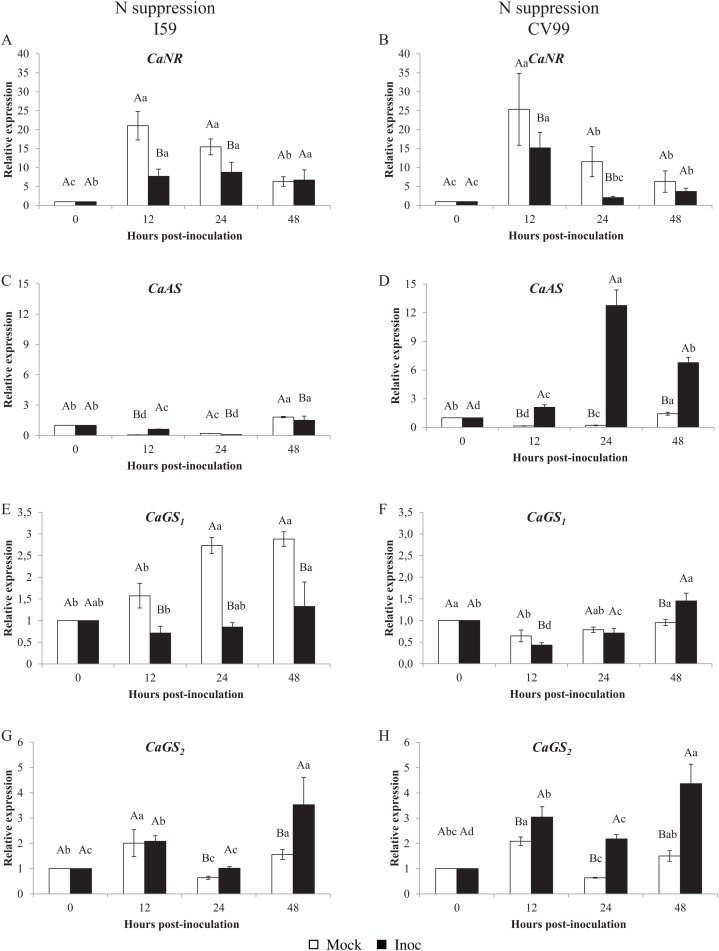
N metabolism gene expression patterns using RT-qPCR analysis for resistant (I59) (A, C, E and G) and susceptible (CV99) (B, D, F and H) genotypes under N suppression at 0, 12, 24 and 48 h post-inoculation with mock and rust inoculated coffee leaves. The vertical bars indicate the values presented by the mean ± standard error (*n* = 9) of N metabolism gene for each time post-inoculation and genotype. Averages followed by the same capital letter, compare differences between mock and rust inoculated coffee leaves at the same HPI, did not differ significantly by the Tukey test (*p* < 0.05). Averages followed by the same lowercase letter, compare differences between the treatments (mock and inoc) at different HPI, did not differ significantly by the Tukey test (*p* < 0.05). *CaGAPDH* was used as reference gene and 0 HPI mock inoculation as calibrator.

Figure 1 shows that the *C. arabica* assimilation genes were differentially regulated according to the resistance to CLR and to N nutritional regimes. As a consequence of CLR inoculation, we observed different expression patterns for all genes between genotypes. The *CaNR* gene had the highest transcriptional level observed in both N conditions (Figs. 1A and 2A). Cultivars I59 and CV99 showed higher levels of *CaNR* transcripts at 12 HPI under N-sufficiency and -suppression (Figs. 1A, 1B, 2A and 2B). However, I59 showed high relative expression values of the *CaNR* gene in comparison to CV99 during N sufficiency (Figs. 1A and 1B), while the opposite pattern was observed during N suppression (Figs. 2A and 2B). The *CaNR* gene transcriptional activity for I59 was up-regulated under *H. vastatrix* infection after 12 HPI (Fig. 1A), while for CV99, it was up-regulated after 12 and 24 HPI (Fig. 1B). Mock and inoculated leaves under N suppression showed similar transcriptional activity profiles of *CaNR* for both genotypes (Figs. 2A and 2B). In all time-points analyzed, *CaNR* gene expression profiles were always higher in the mock samples (white bars) than in the inoculated leaves (black bars) (Figs. 2A and 2B).

The *CaAS* transcriptional profiles were similar for leaves under mock and fungus inoculation treatments for both cultivars under N sufficiency (Figs. 1C and 1D). The *CaAS* transcript levels were higher in mock inoculation compared to leaves infected with *H. vastatrix* at 48 HPI for I59 (Fig. 1C) and at 12, 24 and 48 HPI for CV99 (Fig. 1D). On the other hand, under N suppression, *CaAS* gene transcriptional activity from CV99 was up-regulated after 12 HPI in leaves infected with CLR, with the highest transcript level detected at 24 HPI (Fig. 2D); the expression values remained high at 48 HPI. Additionally, the CV99 genotype increased the transcriptional level of *CaAS* gene 10 fold in relation to the resistant genotype in response to CLR disease (Figs. 2C and 2D). The *CaAS* gene transcriptional activity from the I59 genotype was up-regulated under fungus attack after 12 HPI (Fig. 2C).

The *CaGS*_*2*_ gene expression was highly similar to the *CaAS* gene transcriptional profile (Figs. 1 and 2). Under N sufficiency, rust infection did not influence *CaGS*_*2*_ expression for both genotypes, resulting in higher peaks for mock samples or showing no statistical differences between post-inoculation times (Figs. 1G and 1H). Under N suppression, I59 showed transcriptional peaks at 24 and 48 HPI in response to CLR (Fig. 1G), and CV99 increased transcriptional levels for *CaGS*_*2*_ at all post-inoculation times in response to the fungus (Fig. 1H).

The *CaGS*_*2*_ transcriptional levels were higher than those of *CaGS*_*1*_ in both N nutritional regimes and genotypes (Figs. 1 and 2). Nitrogen suppression resulted in an increased relative expression for *CaGS*_*1*_ at 48 HPI for CV99 (Fig. 2F); I59 showed increased transcripts at all post-inoculation times for mock inoculation (Fig. 2E). However, under N sufficiency, I59 showed increased transcripts rates of *CaGS*_*1*_ at the initial and final moments of pathogen attack (12 and 48 HPI, Fig. 1E). These results show that CV99 did not present statistical differences between mock and inoculated treatments at all post-inoculation times (Fig. 1F).

### Glutamine synthetase in vitro activity

**Figure 3:**
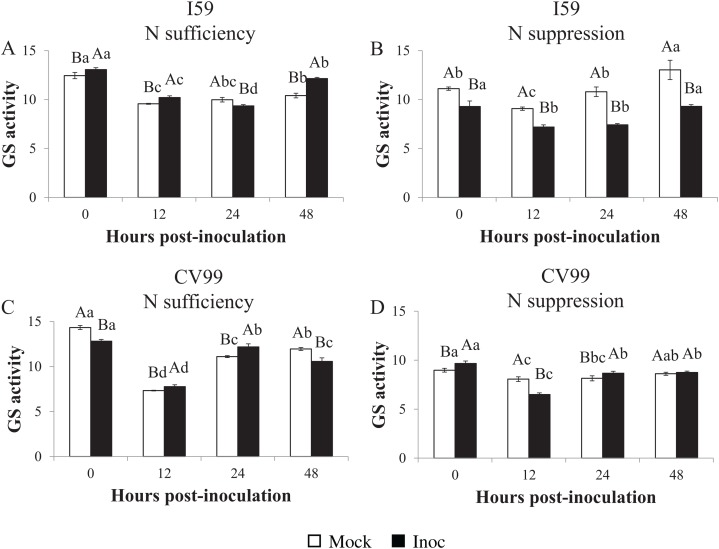
Glutamine synthetase in vitro activity in leaves of resistant (I59) (A and B) and susceptible (CV99) (C and D) coffee genotypes under N sufficiency (A and C) and N suppression (B and D) conditions with mock and rust inoculated coffee leaves. The vertical bars indicate the values presented by the mean ± standard error (*n* = 3) of GS in vitro activity for each hour post-inoculation (HPI) and genotype. Averages followed by the same capital letter, compare differences between mock and rust inoculated coffee leaves at the same HPI, did not differ significantly by the Tukey test (*p* < 0.05). Averages followed by the same lowercase letter, compare differences between the treatments (mock and inoc) at different HPI, did not differ significantly by the Tukey test (*p* < 0.05).

Determination of the GS in vitro activity showed similar transcriptional patterns for *CaGS*_*1*_ in both N nutritional regimes (Figs. 1–3). Nitrogen suppression resulted in a decreased in vitro activity of GS, except for I59 mock-inoculated samples at 24 and 48 HPI and for CV99 at 12 HPI, where GS activity increased (Figs. S4A and S4C). The GS in vitro activity was higher in rust-inoculated samples for both genotypes under N sufficiency (Figs. S4B and S4D).

Accordingly, under N sufficiency, GS activities in response to CLR were influenced at 0, 12 and 48 HPI for I59 (Fig. 3A) and at 12 and 24 HPI for CV99 (Fig. 3C). Based on Fig. 3B under N suppression, rust infection did not influence the GS activities for I59, resulting in higher peaks for mock samples at all post-inoculation times. The cultivar CV99 showed transcriptional peaks at 0 and 24 HPI in response to CLR (Fig. 3D). According to these results, there was an increase of GS in vitro activity under N sufficiency for the I59 genotype in response to the fungus inoculation and a rapid GS activity for CV99 coffee plants under N sufficiency.

### Nitrate reductase in vitro activity

**Figure 4:**
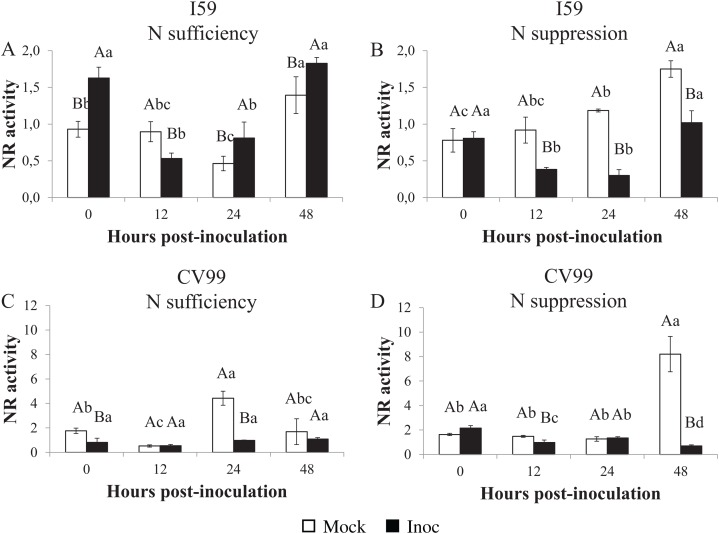
Nitrate reductase in vitro activity in coffee leaves of resistant (I59) (A and B) and susceptible (CV99) (C and D) coffee genotypes under N sufficiency (A and C) and N suppression (B and D) conditions with mock and rust inoculated coffee leaves. The vertical bars indicate the values presented by the mean ± standard error (*n* = 3) of NR in vitro activity for each hour post-inoculation (HPI) and genotype. Averages followed by the same capital letter, compare differences between mock and rust inoculated coffee leaves at the same HPI, did not differ significantly by the Tukey test (*p* < 0.05). Averages followed by the same lowercase letter, compare differences between the treatments (mock and inoc) at different HPI, did not differ significantly by the Tukey test (*p* < 0.05).

The NR activities varied under different N nutritional regimes for the I59 genotype (Figs. 4A and 4B), with high activity levels at 0, 24 and 48 HPI in response to CLR under N sufficiency (Fig. 4A; Fig. S5B). Under N suppression (Fig. 4B), rust infection did not influence the NR activities for I59, resulting in higher peaks for mock samples at all post-inoculation times; the same was observed for *CaNR* gene transcripts. In this study, we obtained an increase of the NR activity in infected leaves of the genotype I59 under N sufficiency (Fig. 4A).

Coffee plants of the CV99 genotype, under N sufficiency, showed a transcriptional peak value at 24 HPI for mock samples (Fig. 4C; Fig. S5C). Figure 4D and Figure S5C illustrate the NR activity peak observed at 48 HPI for mock samples of CV99 under N suppression. These results lead us to infer that there was a rapid NR biosynthesis when N was available for CV99 coffee plants, but when it was suppressed, there was a delay in NR production.

### Chlorophyll levels

In both genotypes, the chlorophyl concentration in leaves was influenced by N suppression and by rust inoculation. As shown in Table S1, for both nutritional regimens, chlorophyl *a* concentration was higher compared to chlorophyl *b*.

**Figure 5:**
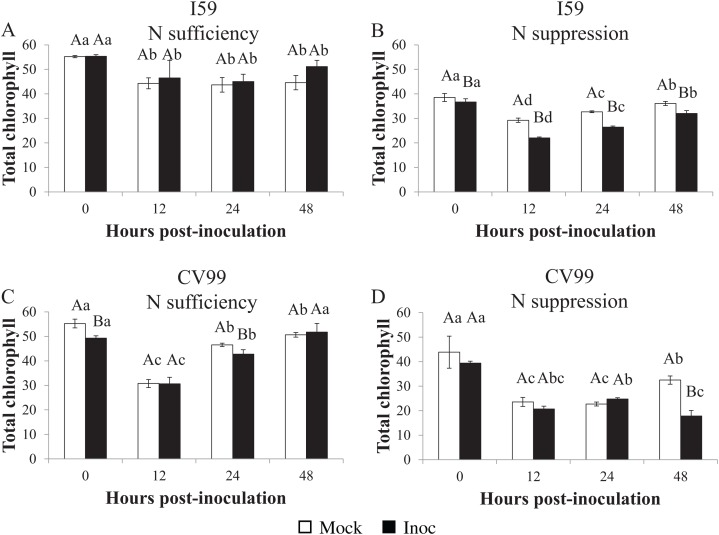
Concentration of total chlorophyl in resistant (I59) (A and B) and susceptible (CV99) (C and D) coffee genotypes under N sufficiency (A and C) and N suppression (B and D) conditions with mock and rust inoculated coffee leaves. The vertical bars indicate the values presented by the mean ± standard error (*n* = 3) of NR in vitro activity for each hour post-inoculation (HPI) and genotype. Averages followed by the same capital letter, compare differences between mock and rust inoculated coffee leaves at the same HPI, did not differ significantly by the Tukey test (*p* < 0.05). Averages followed by the same lowercase letter, compare differences between the treatments (mock and inoc) at different HPI, did not differ significantly by the Tukey test (*p* < 0.05).

The total chlorophyl concentration in coffee leaves under N sufficiency differed between the different post-inoculation times (Figs. 5A and 5C). For I59, the concentration decreased (Fig. 5A), while for CV99, it mainly decreased at 12 HPI, but recovered at 48 HPI (Fig. 5C). Furthermore, in the mock inoculation treatment for both genotypes, the total levels of chlorophyl, chlorophyl *a*, and chlorophyl *b* were similar in the beginning (0 HPI), that is, 55, 30 and 25 µg/mL, as shown in Tables S1A and S1B, respectively.

Therefore, according to our results under N suppression, the total chlorophyl concentrations decreased significantly in comparison to the treatment with N sufficiency (Figs. 5B and 5D). In both genotypes, a reduction in the chlorophyl concentration after 12 HPI occurred for mock- and for rust-inoculated leaves (Figs. 5B and 5D), but at 48 HPI, the concentration was further reduced for CV99 in response to inoculation (Fig. 5D), suggesting that the degradation of cellular components was accelerated by the disease and, consequently, by leaf senescence under N suppression.

## Discussion

As reviewed by Farjad et al. (2018), the availability of N influences plant–pathogen interactions. These same authors also reinforce the idea that, according to the availability and form of N, these factors may be determinant for the effect of N supply on disease severity. Molecularly, some genes involved in N metabolism in response to pathogen infection exhibit a pattern of modulation according to the pathogen lifestyle (biotrophic, hemi-biotrophic, or necrotrophic) and types of plant–pathogen interactions (compatible or incompatible) (Fagard et al., 2014). Rust fungi are obligate parasites that establish a biotrophic interaction with their plant host. Nitrogen metabolism-related gene modulation is also influenced by genotype efficiency in N assimilation and/or remobilization (Thalineau et al., 2018). Accordingly, the N source has also an impact on the interaction between plant and fungi, including their gene expression patterns (Koegel et al., 2015).

Our results show that CLR induced distinct responses in the transcriptional levels of N-metabolism genes evaluated under different N nutritional regimes and genotypes. Under N sufficiency conditions, I59 showed higher transcript levels for *CaNR* and *CaGS*_*1*_ in the initial moment of pathogen inoculation (12 HPI) (Figs. 1A and 1E), suggesting a different capacity of N assimilation in the resistant genotype, which may result in the improvement of plant resistance against CLR. In a previous study, Horst et al. (2010) observed a substantial increase in glutamine and asparagine contents, while enzyme activities involved in primary N assimilation were reduced by 50% in infected plants compared to mock controls in a biotrophic fungus–maize interaction.

In contrast, *H. vastatrix* did not influence the expression patterns for *CaNR* and *CaGS*_*1*_ for both genotypes under N suppression, except for CV99 at 48 HPI for *CaGS*_*1*_ (Figs. 1A, 1B, 1E and 1F). In addition, CLR activated the expression of *CaNR* also for CV99. These results suggest that N remobilization was influenced by the pathogens and can occur to redirect plant nutrients, probably for pathogen colonization of the susceptible genotype.

Regarding GS genes, under N sufficiency, *CaGS*_*1*_ increased its transcriptional levels at 12 and 48 HPI for the resistant genotype (Fig. 1E). Here, GS may facilitate N remobilization, thereby reducing N availability to the pathogen, as a defense mechanism. Conversely, the levels of *CaGS*_*2*_ were increased for both genotypes under N suppression (Figs. 2G and 2H). This may be due to the progression of the fungal infection and to N deficiency induction, leading to N remobilization. In a previous study, *GS1* could function as a metabolic defense-gene, in which the gene was detected very rapidly (2 h after inoculation) in response to *Pseudomonas syringae* pv. *syringae* in tobacco (Pageau et al., 2006). Tavernier et al. (2007) suggest that bean leaves responded to pathogen (*Colletotrichum lindemuthianum*) recognition by inducing *GS1α*, which resulted in glutamine accumulation in infected leaf tissues.

In our study, in vitro GS activity decreased with N suppression and CLR infection (Figs. S4B and S4D). According to Tavernier et al. (2007), the total GS activity decreased with the progression of the disease in common bean, suggesting that primary N assimilation through the GS2/GOGAT pathway was affected by pathogen infection and that N remobilization was induced. In the model plant *A. thaliana*, low N availability severely compromises the plant’s ability to express pathogen-induced resistance (Dietrich, Ploss & Heil, 2005), presenting a delay in the transcript level of defense genes and a decrease in the levels of corresponding enzymes.

As such, *CaAS* transcripts were influenced by CLR under N suppression, with a significant increase in the mRNA levels for I59 at 12 HPI and for CV99 at all post-inoculation times (Figs. 2C and 2D). The gene AS is highly expressed in situations of N remobilization/recycling (Fagard et al., 2014). Previous studies have shown that *AS* is modulated by interactions with pathogens, such as in *Arabidopsis* (Ward et al., 2010), tomato (Olea et al., 2004; Seifi et al., 2014), and pepper (Hwang, An & Hwang, 2011). Moreover, Fernandez et al. (2012) have demonstrated that *AS* is one of the most induced genes in *C. arabica* leaves infected with rust fungi after 21 days post-inoculation. In this context, we suggest that the rapidly and highly induced *AS* in the susceptible genotype may play a dual role by redirecting nutrients for pathogen colonization and by accelerating plant senescence under N suppression. Consistent with these results, Seifi et al. (2014) hypothesize that rapid activation of *AS* in susceptible tomato cultivars may promote *Botrytis cinerea* pathogenesis by providing a rich source of N for the pathogen. In the same way, *AS1* was strongly up-regulated in response to pathogen attacks in *Arabidopsis* leaves, suggesting that *AS1* transcriptional activity is facilitated by a signaling mechanism that is activated by the pathogen itself in order to gain access to valuable amino acids (Ward et al., 2010).

## Conclusions

In summary, in this study, we observed that N assimilation genes were modulated by CLR in a genotype-specific way, prospecting that these genes may be involved in the resistance response of coffee plants against *H. vastatrix*. The occurrence of N remobilization was dependent on mineral N availability. Our results open new perspectives of studies to improve our knowledge about physiological and molecular responses of coffee plants under different mineral N availabilities and under biotic stress for two contrasting genotypes.

## Supplemental Information

10.7717/peerj.8320/supp-1 Supplemental Information 1 Differential nutritional regimes for I59 (A) and CV99 (B) coffee genotypes. Yellow labels refer to plants in N suppression condition (0 μm NH_4_NO_3_) and purple labels refer to plants in N sufficiency condition (5333 μm NH_4_N.

10.7717/peerj.8320/supp-2 Supplemental Information 2 *Hemileia vastatrix* symptoms on susceptible genotype coffee leaves (CV99) to validate the pathogen infection in the differential nutritional pattern and CLR interaction (A and B).

10.7717/peerj.8320/supp-3 Supplemental Information 3 Hypersensitivity reaction with small chlorotic spots associated with punctiform swellings in resistant coffee genotype (I59) on the adaxial (A) and abaxial (B) surfaces in the differential nutritional pattern and CLR interaction.

10.7717/peerj.8320/supp-4 Supplemental Information 4 Glutamine synthetase in vitro activity in leaves of resistant (I59) (A and B) and susceptible (CV99) (C and D) coffee genotypes with mock (A and C) and rust inoculated (B and D) coffee leaves under N sufficiency and N suppression conditions. The vertical bars indicate the values presented by the mean ± standard error (*n* = 3) of GS in vitro activity for each hour post-inoculation (HPI) and genotype. Averages followed by the same capital letter, compare differences between mock and rust inoculated coffee leaves at the same HPI, did not differ significantly by the Tukey test (*p* < 0.05). Averages followed by the same lowercase letter, compare differences between the treatments (mock and inoc) at different HPI, did not differ significantly by the Tukey test (*p* < 0.05).

10.7717/peerj.8320/supp-5 Supplemental Information 5 Nitrate reductase in vitro activity in leaves of resistant (I59) (A and B) and susceptible (CV99) (C and D) coffee genotypes with mock (A and C) and rust inoculated (B and D) coffee leaves under N sufficiency and N suppression conditions. The vertical bars indicate the values presented by the mean ± standard error (*n* = 3) of NR in vitro activity for each hour post-inoculation (HPI) and genotype. Averages followed by the same capital letter, compare differences between mock and rust inoculated coffee leaves at the same HPI, did not differ significantly by the Tukey test (*p* < 0.05). Averages followed by the same lowercase letter, compare differences between the treatments (mock and inoc) at different HPI, did not differ significantly by the Tukey test (*p* < 0.05).

10.7717/peerj.8320/supp-6 Supplemental Information 6 Concentration of total chlorophyl, *a* and *b* in resistant (I59) and susceptible (CV99) genotypes under N sufficiency (A and B) and N suppression (C and D) conditions with mock and *Hemileia vastatrix* inoculation. Data are expressed as the mean ± standard error (*n* = 3) of total chlorophyl concentration, *a* and *b* for each post-inoculation time and genotype. *Averages followed by the same lowercase letter in the column did not differ significantly by the Tukey test (*p* < 0.05).

10.7717/peerj.8320/supp-7 Supplemental Information 7 Raw data from RT-qPCR and biochemical analyses.

## Additional Information and Declarations

### Data Availability

The following information was supplied regarding data availability: All datasets are available in the Supplemental Materials.
