# Supplementary figures and images for: Transcriptional patterns of Coffea arabica L. nitrate reductase, glutamine and asparagine synthetase genes are modulated under nitrogen suppression and coffee leaf rust
Source: PeerJ. 2020 Jan 3;8:e8320. doi: 10.7717/peerj.8320 (PMC6944126; doi:10.7717/peerj.8320)

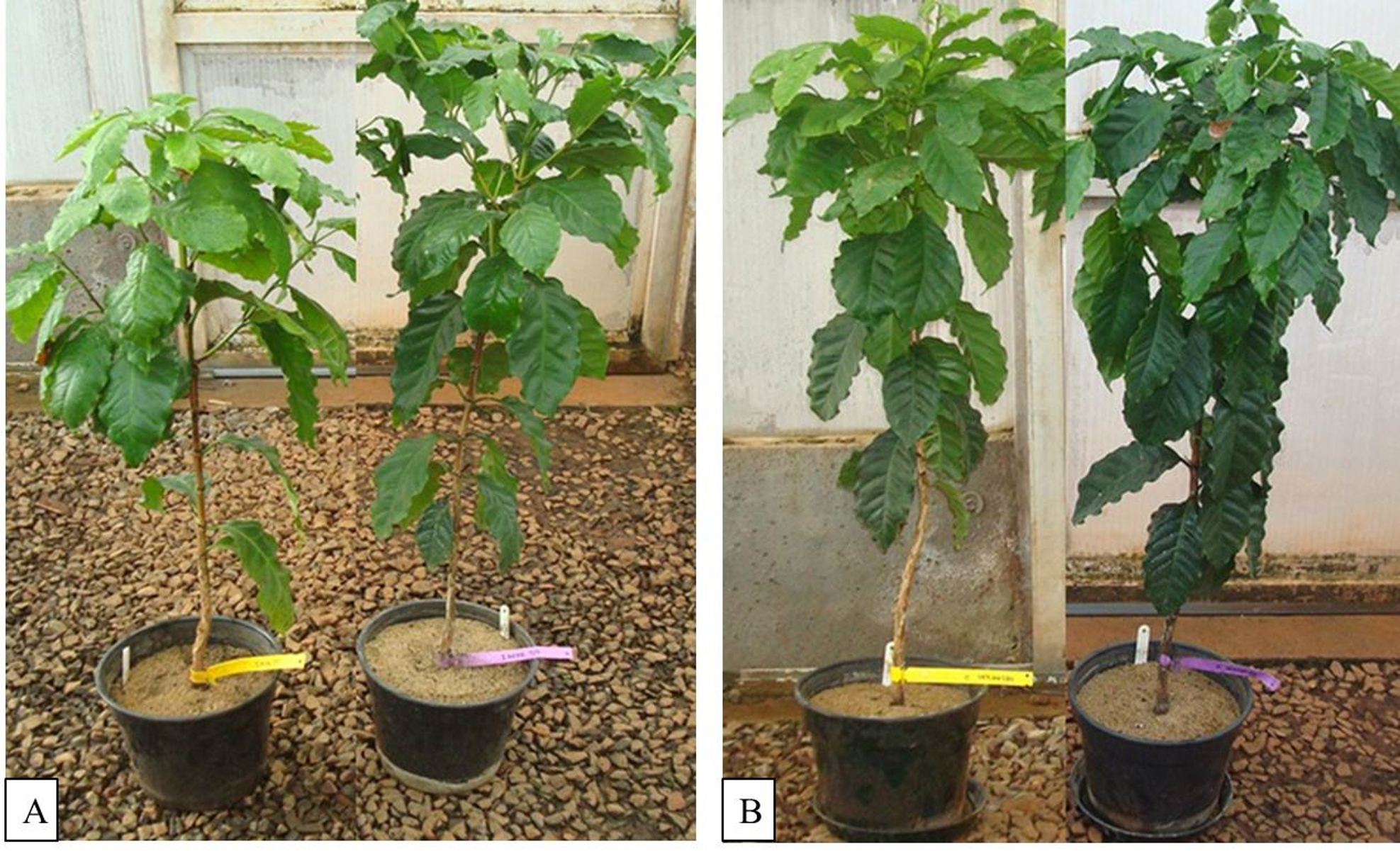

Supplement: Supplemental Information 1 [file peerj-08-8320-s001.jpg]

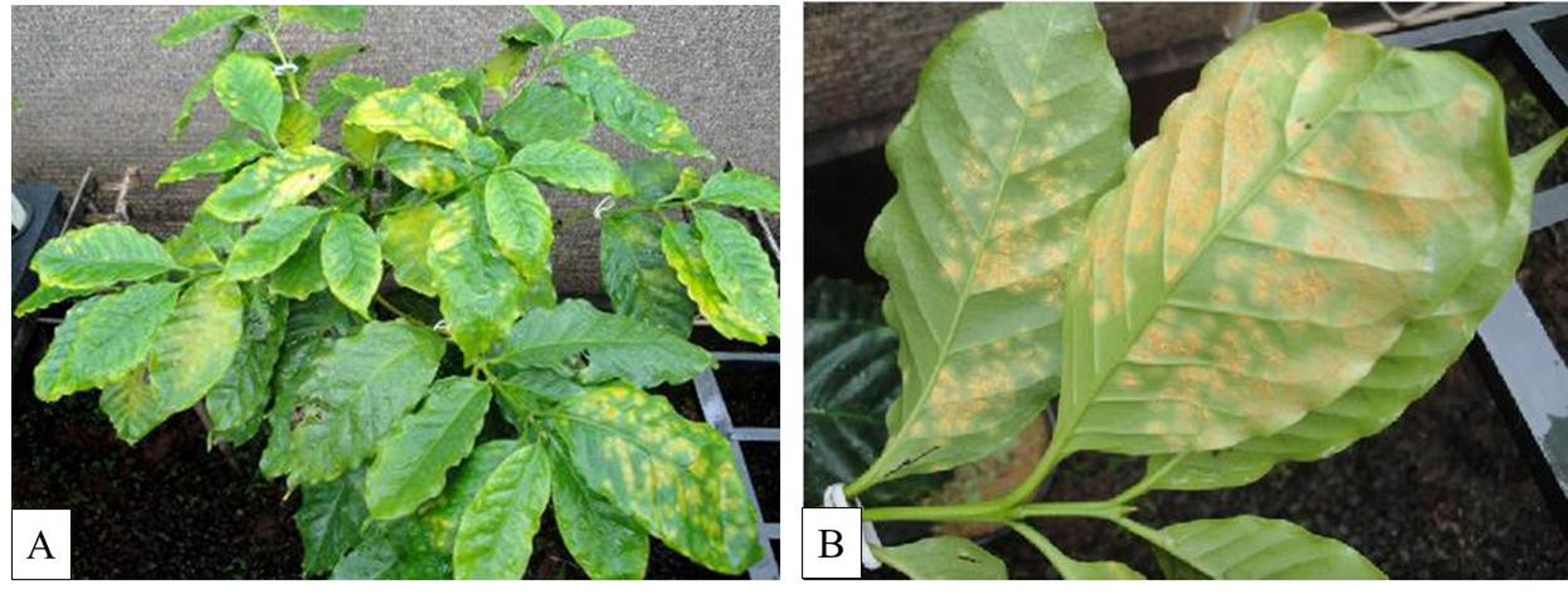

Supplement: Supplemental Information 2 [file peerj-08-8320-s002.jpg]

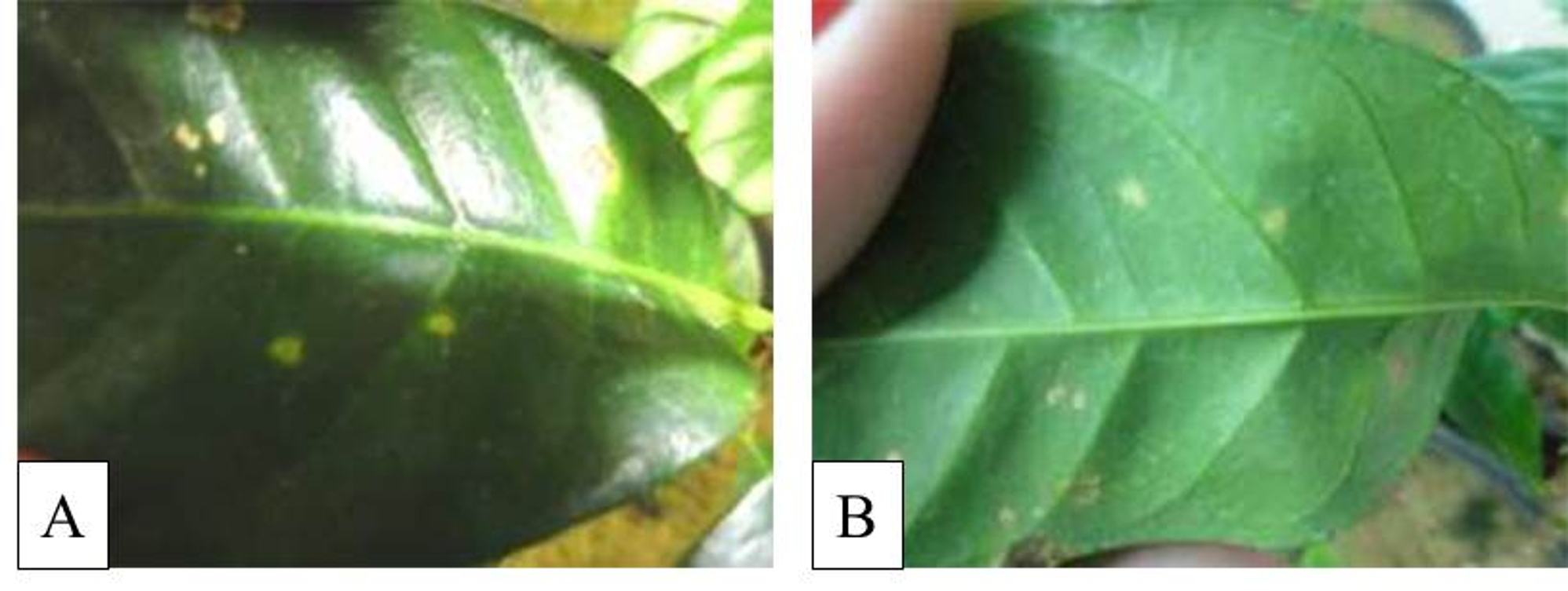

Supplement: Supplemental Information 3 [file peerj-08-8320-s003.jpg]

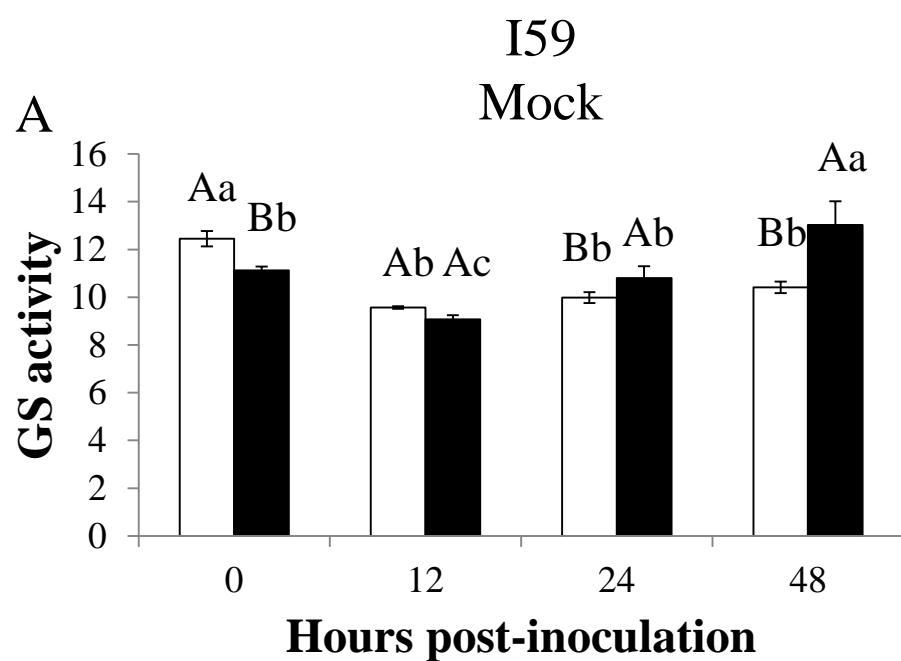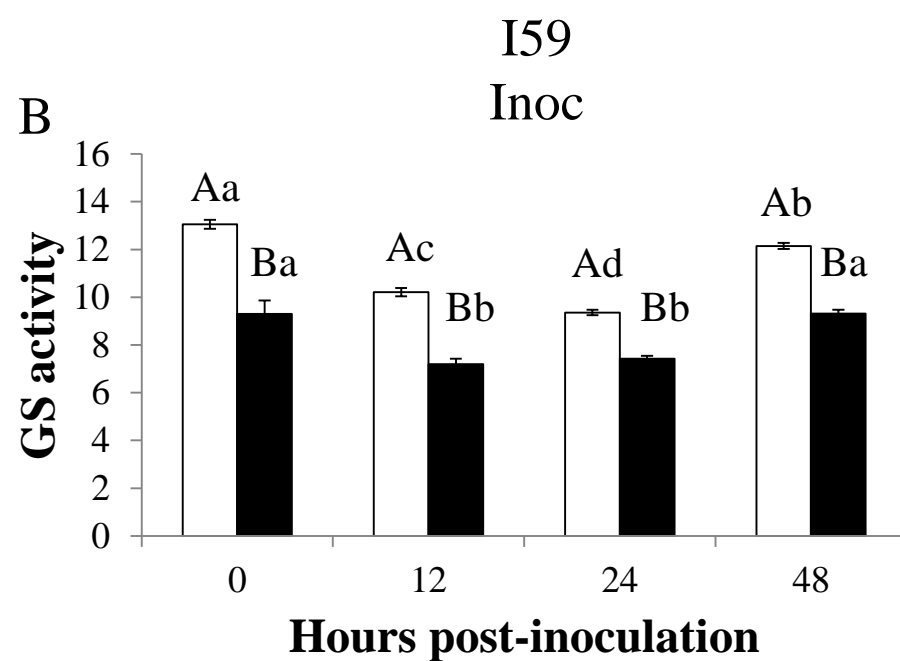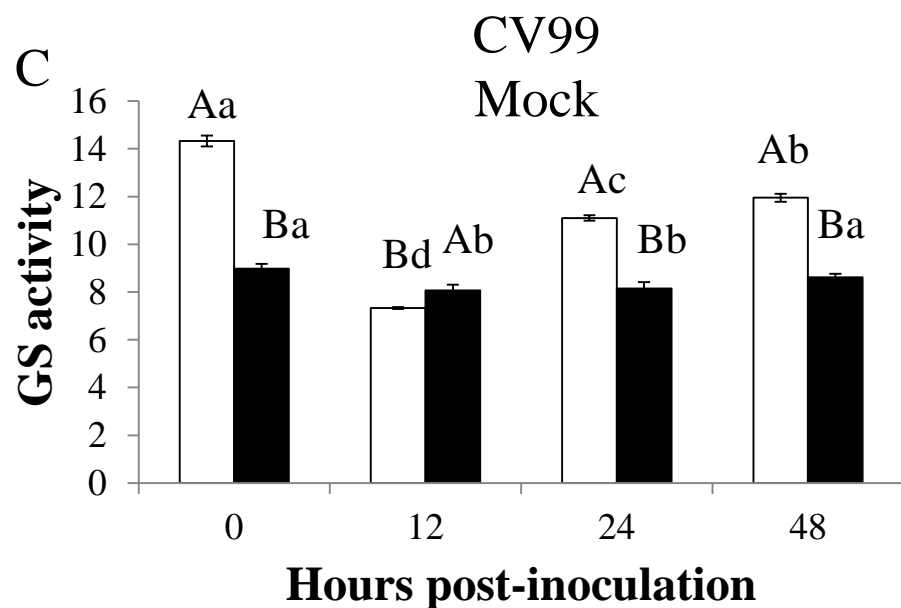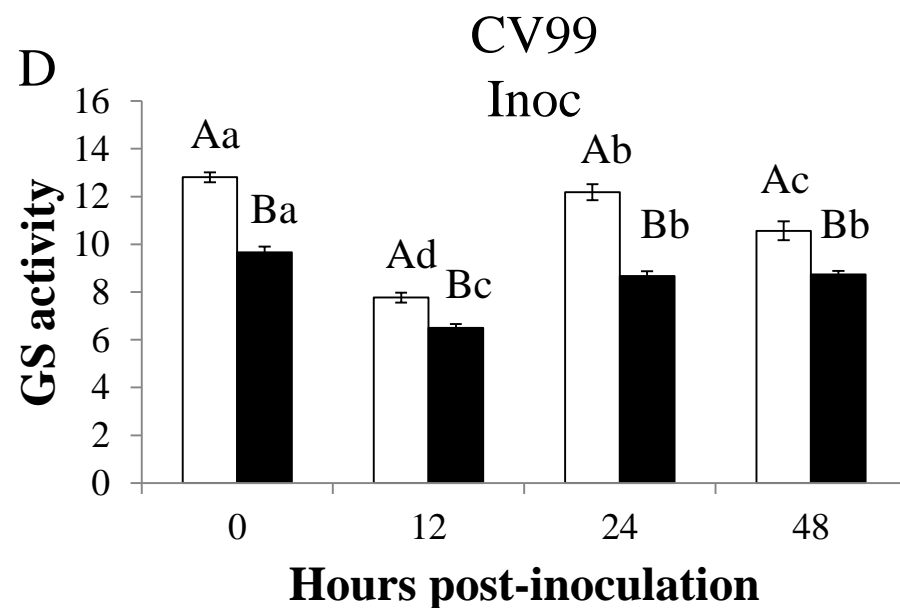

□ N sufficiency    ■ N suppression

Supplement: Supplemental Information 4 — The vertical bars indicate the values presented by the mean ± standard error (n = 3) of GS in vitro activity for each hour post-inoculation (HPI) and genotype. Averages followed by the same capital letter, compare differences between mock and rust inoculated coffee leaves at the same HPI, did not differ significantly by the Tukey test (p < 0.05). Averages followed by the same lowercase letter, compare differences between the treatments (mock and inoc) at different HPI, did not differ significantly by the Tukey test (p < 0.05). [file peerj-08-8320-s004.pdf]

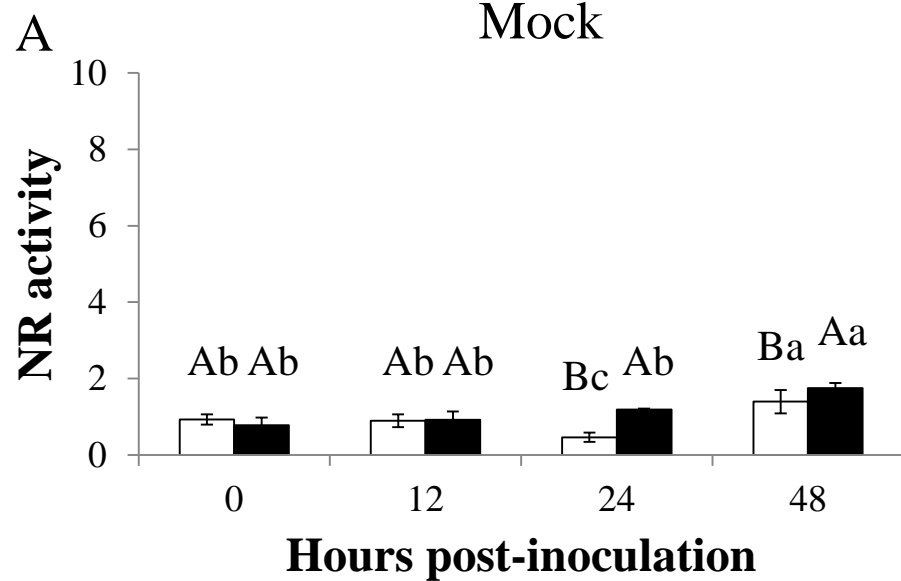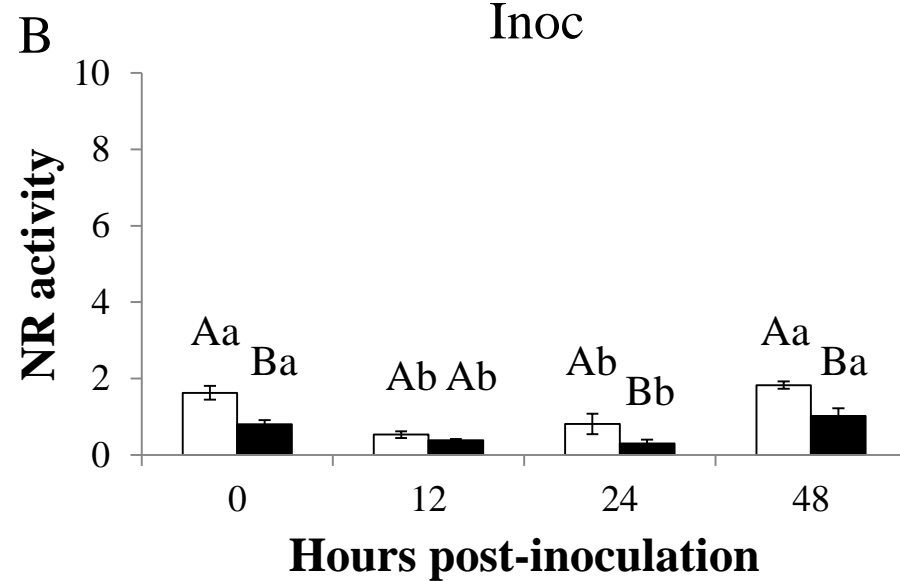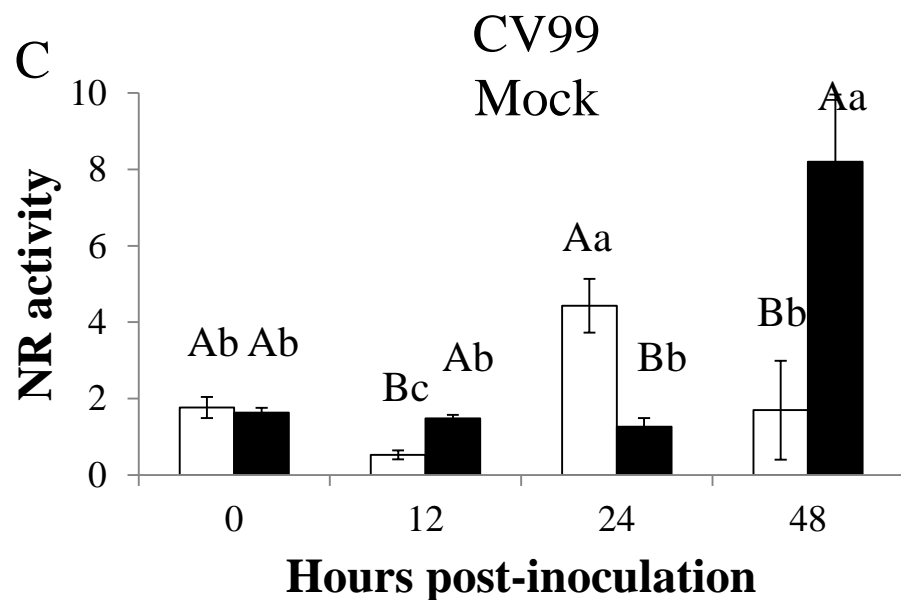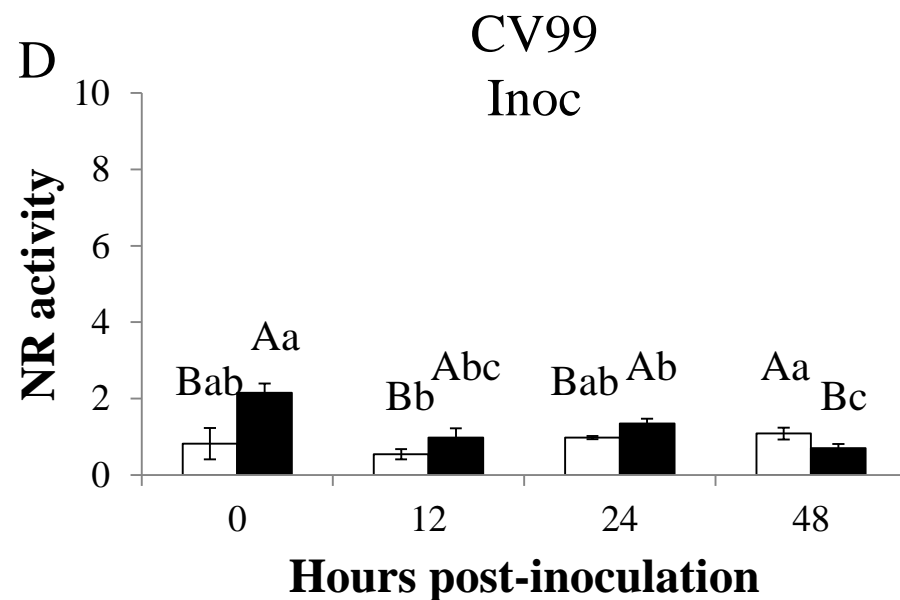

□ N sufficiency    ■ N suppression

Supplement: Supplemental Information 5 — The vertical bars indicate the values presented by the mean ± standard error (n = 3) of NR in vitro activity for each hour post-inoculation (HPI) and genotype. Averages followed by the same capital letter, compare differences between mock and rust inoculated coffee leaves at the same HPI, did not differ significantly by the Tukey test (p < 0.05). Averages followed by the same lowercase letter, compare differences between the treatments (mock and inoc) at different HPI, did not differ significantly by the Tukey test (p < 0.05). [file peerj-08-8320-s005.pdf]
