# Supplementary material for: Transcriptional patterns of Coffea arabica L. nitrate reductase, glutamine and asparagine synthetase genes are modulated under nitrogen suppression and coffee leaf rust
Source: PeerJ. 2020 Jan 3;8:e8320. doi: 10.7717/peerj.8320 (PMC6944126; doi:10.7717/peerj.8320)
Supplement: Supplemental Information 6 — Data are expressed as the mean ± standard error (n = 3) of total chlorophyl concentration, a and b for each post-inoculation time and genotype. *Averages followed by the same lowercase letter in the column did not differ significantly by the Tukey test (p < 0.05). [file peerj-08-8320-s006.docx]

| A | IAPAR 59- N sufficiency | | | | | | |
| --- | --- | --- | --- | --- | --- | --- | --- |
| Time post-inoculation (h) | Mock inoculation (μg/mL) | | |  | Rust inoculation (μg/mL) | | |
|  | Chlorophyll *a** | Chlorophyll *b** | Total chlorophyll* |  | Chlorophyll *a** | Chlorophyll *b** | Total chlorophyll* |
| 0 | 30.5 ± 0.2 a | 24.7 ± 0.3 ab | 55.2 ± 0.4 a |  | 30.3 ± 0.4 a | 25.1 ± 0.8 a | 55.4 ± 0.5 a |
| 12 | 29.3 ± 0.7 a | 15.0 ± 1.2 c | 44.3 ± 1.8 b |  | 29.3 ± 0.8 a | 17.2 ± 5.1bc | 46.5 ± 5.9ab |
| 24 | 28.9 ± 0.9 a | 14.7 ± 1.5 c | 43.7 ± 2.4 b |  | 29.5 ± 0.7 a | 15.6 ± 1.7 c | 45.1 ± 2.4 b |
| 48 | 29.3 ± 0.7 a | 15.3 ± 1.6 c | 44.6 ± 2.4 b |  | 30.5 ± 0.1 a | 20.7 ± 2.1 ac | 51.1 ± 2.1 ab |
| B | Catuaí Vermelho IAC 99- N sufficiency | | | | | | |
| Time post-inoculation (h) | Mock inoculation (μg/mL) | | |  | Rust inoculation (μg/mL) | | |
|  | Chlorophyll *a** | Chlorophyll *b** | Total chlorophyll* |  | Chlorophyll *a** | Chlorophyll *b** | Total chlorophyll* |
| 0 | 30.3 ± 0.2 a | 24.9 ± 1.6 a | 55.2 ± 1.5 a |  | 30.4 ± 0.2 a | 18.9 ± 0.7 cd | 49.3 ± 0.8b |
| 12 | 21.9 ± 0.9 b | 8.9 ± 0.4 f | 30.8 ± 1.3 d |  | 21.9 ± 1.5 b | 8.8 ± 0.7 f | 30.7 ± 2.2 d |
| 24 | 29.9 ± 0.2 a | 16.6 ± 0.4de | 46.5 ± 0.6bc |  | 28.5 ± 0.6 a | 14.3 ± 0.9 e | 42.7 ± 1.5 c |
| 48 | 30.4 ± 0.1 a | 20.2 ± 0.8bc | 50.6 ± 0.8ab |  | 28.7 ± 2.6 a | 23.1 ± 0.7 ab | 51.8 ± 2.9 ab |
| C | IAPAR 59- N suppression | | | | | | |
| Time post-inoculation (h) | Mock inoculation (μg/mL) | | |  | Rust inoculation (μg/mL) | | |
|  | Chlorophyll *a** | Chlorophyll *b** | Total chlorophyll* |  | Chlorophyll *a** | Chlorophyll *b** | Total chlorophyll* |
| 0 | 26.3 ± 0.7 a | 12.2 ± 0.7 a | 38.5 ± 1.3 a |  | 25.4 ± 0.6 a | 11.3 ± 0.5 ab | 36.7 ± 1.1 a |
| 12 | 20.8 ± 0.5 c | 8.4 ± 0.3 de | 29.2 ± 0.7 c |  | 15.8 ± 0.3 e | 6.2 ± 0.1 f | 22.0 ± 0.4 e |
| 24 | 22.7 ± 0.4 b | 10.0 ± 0.5 bc | 32.7 ± 0.3 b |  | 18.8 ± 0.3 d | 7.7 ± 0.1 e | 26.4 ± 0.4 d |
| 48 | 25.1 ± 0.5 a | 11.0 ± 0.2ac | 36.1 ± 0.7 a |  | 22.4 ± 0.4bc | 9.7 ± 0.7 cd | 32.0 ± 0.9 b |
| D | Catuaí Vermelho IAC 99- N suppression | | | | | | |
| Time post-inoculation (h) | Mock inoculation (μg/mL) | | |  | Rust inoculation (μg/mL) | | |
|  | Chlorophyll *a** | Chlorophyll *b** | Total chlorophyll* |  | Chlorophyll *a** | Chlorophyll *b** | Total chlorophyll* |
| 0 | 28.6 ± 1.9 a | 12.8 ± 0.2 a | 40.1 ± 0.5 a |  | 27.1 ± 0.4 a | 12.3 ± 0.3 a | 39.4 ± 0.7 a |
| 12 | 16.9 ± 1.1 c | 6.7 ± 0.4 cd | 23.6 ± 1.5 cd |  | 15.0 ± 0.7 cd | 5.7 ± 0.3 de | 20.7 ± 1.0 de |
| 24 | 16.1 ± 0.4 cd | 6.6 ± 0.3 cd | 22.7 ± 0.7 cd |  | 17.6 ± 0.4 c | 7.2 ± 0.1 c | 24.8 ± 0.4 c |
| 48 | 23.0 ± 0.9 b | 9.5 ± 0.5 b | 32.5 ± 1.4 b |  | 12.8 ± 1.4 d | 5.0 ± 0.5 e | 17.8 ± 1.8 e |
